# New Polycationic Arabinogalactan Derivatives with the CHPTAC System: Structure, Properties and Antioxidant Activity

**DOI:** 10.3390/polym18020148

**Published:** 2026-01-06

**Authors:** Maria V. Sereda, Yuriy N. Malyar, Valentina S. Borovkova, Alexander S. Kazachenko

**Affiliations:** 1 Institute of Chemistry and Chemical Technology, Krasnoyarsk Science Center, Siberian Branch Russian Academy of Sciences, Akademgorodok 50/24, Krasnoyarsk 660036, Russia; 2 School of Non-Ferrous Metals, Siberian Federal University, Pr. Svobodny 79, Krasnoyarsk 660041, Russia; 3 Institute of Chemical Technologies, Reshetnev Siberian State University of Science and Technology, Mira St. 82, Krasnoyarsk 660049, Russia

**Keywords:** arabinogalactan, CHPTAC, 2,3-epoxypropyltrimethylammonium chloride (EPTAC), quaternization, quaternary ammonium groups, cationic esterifying agent

## Abstract

Cationic arabinogalactan (AG) derivatives with a degree of substitution (0.02–0.19) containing quaternary ammonium groups were prepared by reaction of the etherification of (3-Chloro-2-hydroxypropyl)-trimethylammonium chloride (CHPTAC), catalyzed by an aqueous solution of sodium hydroxide. The effect of etherification was assessed by the degree of substitution (DS). The DS values of the AG samples were controlled by the varied pH of the reaction mixture from 10 to 12 and the duration of the process quaternization (2, 18, 24, 30 and 72 h). In comparison, the quaternized samples of the AG were characterized by physicochemical research methods, such as elemental analysis, gel permeation chromatography (GPC), Fourier Transform Infrared (FTIR), and ^1^H nuclear magnetic resonance (NMR) spectroscopy, and thermogravimetric analysis (TGA). Furthermore, the improved antioxidant capacity of the quaternized AGs was evaluated using the 1,1-diphenyl-2-picrylhydrazyl (DPPH) free radical scavenging assay. It was found that the most favorable conditions for the quaternization process were pH = 12, duration and temperature of the process of 31.6 h and 50 °C, respectively. The esterification reaction was accompanied by hydrolysis side reactions at a longer process.

## 1. Introduction

The “polysaccharides” is a class of common natural macromolecular polymers that are obtained from renewable raw material sources, such as algae, plants, and microorganisms [1]. Plant polysaccharides are the most promising group of substances due to their widespread occurrence, bioavailability, biodegradability, and non-toxicity [2], and the possibility of their participation in complexation reactions [3,4]. A wide range of sought-after properties allows the use of polysaccharides to create various materials, such as delivery systems for medicinal substances [5,6], coagulants [1,7], biosorbents [4,8] and organic gels [9,10].

Arabinogalactan of the larch (*Larix sibirica* L.) is one of the unique heteropolysaccharides [11], which has a number of beneficial properties due to its highly branched structure. The main chain of the arabinogalactan macromolecule consists of galactose units linked by β-(1→3) glycosidic bonds, and the side chains with β-(1→6) bonds are made up of galactose and arabinose units, single arabinose units, and uronic acids, mainly glucuronic acid [12]. The ratio of galactose and arabinose units is approximately 6:1, with 1/3 of the arabinose units in the pyranose form and 2/3 in the furanose form [13].

Arabinogalactan is a water-soluble polysaccharide, which is of great interest for many applications, such as coatings, personal care products, food, and beverages that contain polysaccharides dissolved in aqueous or organic solvents [14]. This polysaccharide is a source of soluble dietary fiber [15], and also has anti-allergenic [16,17], immunomodulatory [17,18] and anti-inflammatory effects [13], allowing it to be used in the medical field as a substance delivery system [18]. In addition, arabinogalactan can be considered as an alternative to gum arabic, guar gum, and starch in various cosmetic and pharmaceutical products. But its use as a chelating agent is somewhat limited due to its disordered structure and the presence of exclusively monodentate ligands represented by hydroxyl groups, which is functionally insufficient for complex formation [19,20]. This problem can be solved by chemical modification of arabinogalactan, which will improve the complexing properties of the polysaccharide.

Currently, the generally accepted methods for molecular modification of polysaccharides include carboxymethylation [21], phosphorylation [22], sulfonation [23], sulfation [24], methylation [25], quaternization [26], and alkylation [27]. One of the promising methods of modification in polymer chemistry is quaternization [28,29]. This process involves the chemical modification of polysaccharides by introducing quaternary ammonium groups, which have a positively charged nitrogen that forms four bonds. Such modification significantly expands the range of physicochemical properties of polymers, including their solubility, biocompatibility, antimicrobial activity, and ability to form films and gels [30,31]. This is especially relevant for the development of new materials in the fields of medicine, pharmacology, the food industry, and wastewater treatment [28,29,32]. In studies aimed at studying quaternized derivatives of polysaccharides, such substances as starch [33], chitosan [34], cellulose [35,36], xylans [37], dextrin [38], pectin [39] and other biopolymers have already been studied, since this type of polymer modification allows their functionality, while maintaining most of the other properties unchanged [26].

This type of chemical modification of arabinogalactan can lead to an improvement in its adhesive properties and to an increase in the ability to bind with other substances. Due to the fact that the quaternary ammonium built into the polysaccharide during the process is a charged group that can actively interact with other functional groups, the ability of arabinogalactan to enter into intermolecular interactions with other materials, for example, with polyanions, increases. Modified arabinogalactan can find application in the creation of new forms of drugs, as a means of delivering active components, antimicrobial and wound-healing coatings, as well as components of biodegradable plastics and packaging materials [40,41]. For example, in the food industry, it can be used as a food preservative and an antimicrobial agent. Due to their positive charge, quaternized derivatives can exhibit antimicrobial activity by interacting with the negatively charged walls of bacterial cells. This makes them promising for use as natural or modified food preservatives to extend the shelf life of products or in antimicrobial packaging [42]. Modified samples can also be used as stabilizers and emulsifiers. Like natural gum arabic (a type of arabinogalactan), quaternized forms can act as effective stabilizers and emulsifiers in various food systems (e.g., beverages, sauces, spreads), improving their texture and preventing separation [43]. Modified arabinogalactan can be used as a biocompatible and biodegradable polymer for the creation of targeted drug delivery systems. For example, it can serve as a basis for nanoparticles or hydrogels capable of binding therapeutic agents (including anticancer drugs) and providing their controlled release in target tissues or cells [44]. Quaternary derivatives can have improved adhesive properties (the ability to adhere to biological tissues, such as mucous membranes), which is useful in the development of patches, eye drops, or transmucosal delivery systems [45]. (3-Chloro-2-hydroxypropyl)-trimethylammonium chloride is one of the most used agents for the preparation of cationic polysaccharides because it is relatively inexpensive, has low toxicity, is more stable, is commercially available, and is environmentally friendly, converting into an epoxide when exposed to alkaline conditions [26].

Based on the above, the aim of the present study is the functionalization of Siberian larch (*Larix sibirica* L.) arabinogalactan with a CHPTAC-based system.

## 2. Materials and Methods

### 2.1. Synthesis of Polycationic Derivatives of the Arabinogalactan

The raw material used was air-dried arabinogalactan, obtained using a proven method [46] from larch wood (*Larix Sibirica* L.), provided from the collection of the Institute of Chemical Technology of the Siberian Branch of the Russian Academy of Sciences.

**Figure 1:**
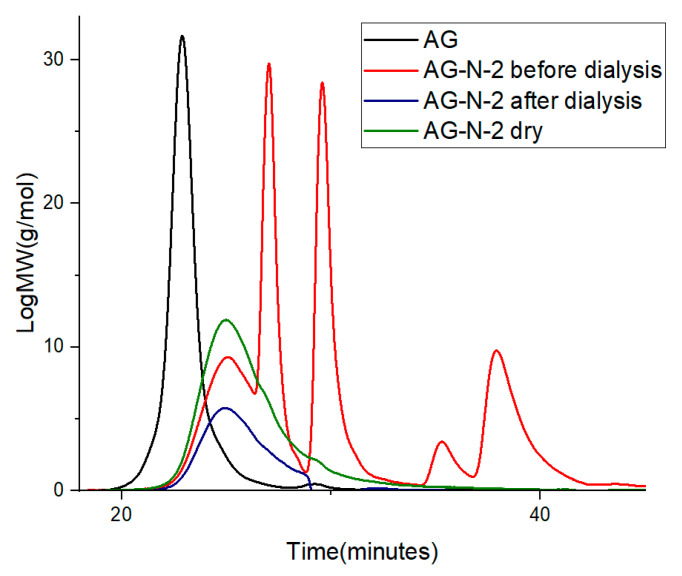
GPC chromatograms of AG and AG-N-2 before and after dialysis and dry sample.

Quaternization of arabinogalactan with the cationic esterifying agent CHPTAC (CAS:3327-22-8, 65 wt% aqueous solution, Macklin Inc., Shanghai, China) was carried out using the methods described in refs. [19,20,21,22,23,24,28], with some modifications. In this work, 50 mL of distilled water, 2.5 g of arabinogalactan, 4.52 mL of CHPTAC, and 0.2 g of NaOH (ECOS-1, Moscow, Russia) were placed in a three-necked flask equipped with a thermometer and a mechanical stirrer for quaternization of arabinogalactan. The resulting mixture was heated with vigorous stirring to 50 °C for different process durations (2–72 h). The pH of the system during the reaction was adjusted by adding 5 M NaOH solution (Figure S1). After the specified time, the process was stopped by adding 1 M HCl (Reagent, Samara, Russia) until pH of 7.0 was reached. To purify the product from unreacted compounds and low-molecular-weight substances, dialysis against water was used in a dialysis bag 5015-19 MWCO with a pore size of 3.5 kDa for 24 h, changing the water every hour. The aqueous solution of quaternized arabinogalactan after dialysis was transferred to a Petri dish and dried in a drying oven at a temperature of 60 °C. Based on the chromatograms, the dialysis purification process removes low-molecular-weight impurities and unreacted residues, indicating complete purification of the polysaccharide. The purity of the samples after dialysis was controlled chromatographically (Figure 1).

The purified and dried sample exhibits a single peak shifted toward the lower-molecular-weight region relative to the original compound. The undialyzed sample exhibits additional peaks not present in the native arabinogalactan structure, confirming low-molecular-weight by-products.

### 2.2. Mathematical Process Optimization

The optimal modification mode of arabinogalactan was found using the DOE (Experimental design) block from the Statgraphic Centurion XVI software package [47].

### 2.3. Elemental Analysis

The elemental composition was studied using a Vario EL cube elemental analyzer (ELEMENTAR, Langenselbold, Germany). The determination conditions were as follows: CHNS configuration, sample combustion in the presence of oxygen, followed by gas adsorption separation and detection of combustion products using a thermal conductivity detector. Measurements were conducted in three independent replicates. The degree of substitution was calculated according to Equation (1) [30,48]:

(1) $$DS=\;\frac{158\;\times \;W_{N}}{1400-151.5\;\times \;W_{N}}$$

where *W_N_*—amount of nitrogen (%) determined by elemental analysis.

### 2.4. Molecular Weight Distribution Properties

The molecular weight characteristics of arabinogalactan and its derivatives were determined by gel permeation chromatography once, given the high intrinsic reproducibility of the technique. The analysis was performed using an Agilent 1260 Infinity II Multi-Detector GPC/SEC System chromatograph (Agilent Technologies, Santa Clara, CA, USA) with a refractometer as the main detector. Separation was performed on combined PL Aquagel-OH-30 and PL Aquagel-OH Mixed-M columns using an aqueous solution of 0.1 M NaNO_3_/250 ppm NaN_3_ as the mobile phase. The column was calibrated using polydisperse polyethylene glycol standards (Agilent Technologies, Santa Clara, CA, USA). The eluent flow rate was 1 mL/min. Data collection and processing were performed using the Agilent GPC/SEC MDS version 2.2 software.

### 2.5. Fourier Transform Infrared Spectroscopy

FTIR spectra of the most representative samples of AG and its derivatives were recorded in a single run due to the high intrinsic reproducibility of the method. The work was performed using a Tensor 27 FTIR spectrometer (Bruker, Ettlingen, Germany). Spectral information was processed using the OPUS version 7.5 software. Solid samples for analysis were prepared as tablets in a KBr matrix (2 mg sample/1000 mg KBr).

### 2.6. Nuclear Magnetic Resonance

Nuclear magnetic resonance ^1^H and ^13^C were recorded using a Bruker Avance III 600 spectrometer (Bruker BioSpin, Rheinstetten, Germany). A single-run analysis was performed on samples of AG and its highly substituted quaternized derivative. Before analysis, samples were completely dissolved in D_2_O and placed in a 5 mm NMR tube at room temperature.

### 2.7. Thermogravimetric Analysis

A single-run simultaneous thermal analysis (TG/DTG) was carried out in an argon atmosphere to study the thermal decomposition of AG and its derivatives. The thermogravimetric analysis was carried out in a corundum crucible using an STA 449 F1 Jupiter device (NETZSCH, Selb, Germany) in the temperature range from 30 to 700 °C in an argon flow (the flow rates of the protective and purging gases were 20 and 50 mL/min, respectively). The heating rate was 5, 10, and 20 °C/min. The measurement results were processed using the NETZSCH Proteus Thermal Analysis 5.1.0 software package supplied with the device. The kinetic characteristics and the mechanism of thermal decomposition of the studied samples were determined using isoconversion (integral and differential) methods of kinetic analysis.

### 2.8. Antioxidant Activity

The absorption capacity of DPPH was used to establish the AGs’ antioxidant activity, determined by the method described in [49]. The AGs samples were dissolved in distilled water in concentrations of 0.05, 0.1, 0.5, 2, and 5 mg/mL. In this study, vitamin C (Vc) was used as a control. The experiments were repeated three times, and the values obtained were averaged.

The scavenging ability of DPPH was calculated as Equation (2):

(2) $$\mathrm{DPPH}\;\mathrm{Radical}\;\mathrm{Scavenging}\;\mathrm{Ability}\;(\%)\;=\;(1-(\frac{A_{c}-A_{b}}{A_{Cs}}))\times 100\%$$

where A_C_ is the absorbance of the DPPH solution without a sample, A_S_ is the absorbance of the test sample mixed with the DPPH solution, and A_b_ is the absorption of the test sample without DPPH [50].

## 3. Results and Discussion

### 3.1. Quaternized Arabinogalactan Derivatives

**Figure 2:**
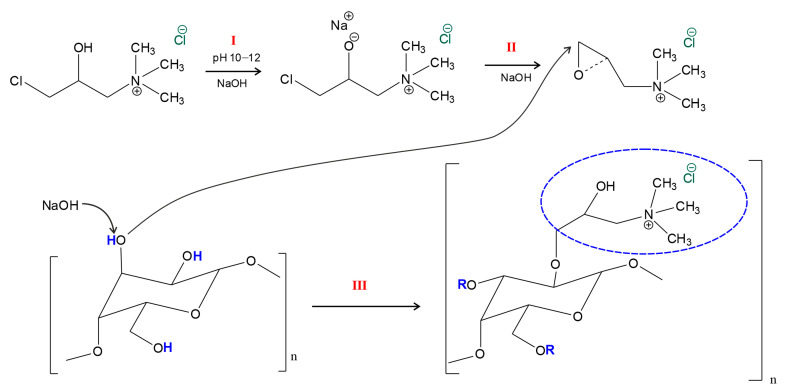
The reaction mechanism of quaternization of the AG unit using the CHPTAC-based system.

In the process of quaternization of AG, amino groups are added to the free hydroxyls of galactose and arabinose units. The proposed mechanism is shown in Figure 2 [26,30,48].

The esterification reaction between the polysaccharide and the CHPTAC system, catalyzed by NaOH, is characterized by nucleophilic substitution in the hydroxyl group of the monosaccharide unit of AG with a quaternary ammonium reagent. Based on the demonstrated reaction mechanism (Figure 2), the quaternization process occurs in three main stages: (I) activation of CHPTAC with an alkaline solution with the formation of an unstable intermediate compound (the rate-limiting step); (II) transformation of the intermediate complex into the reaction agent epoxide EPTAC; interaction of the corresponding epoxide with the hydroxides of the polysaccharide unit.

The NaOH-catalyzed etherification reaction between a polysaccharide and the CHPTAC system is characterized by nucleophilic substitution at the hydroxyl group of the monosaccharide unit AG with a quaternary ammonium reagent. Based on the demonstrated reaction mechanism (Figure 2), the quaternization process occurs in three main steps: (I) the activation of CHPTAC with an alkaline solution to form an unstable intermediate (rate-limiting step), where hydroxide ions catalyze the deprotonation of the secondary hydroxyl group of the reagent to form a metastable alkoxide anion; (II) intramolecular cyclization of the intermediate, which is accompanied by the elimination of the chloride ion and closure of the oxirane ring, resulting in a quantitative conversion of CHPTAC to the reactive epoxide form, EPTAC; (III) the activation of the AG hydroxyl groups and subsequent nucleophilic attack by the epoxide intermediate. In the presence of an alkaline excess, the hydroxyl groups of the AG monosaccharide units—primarily the primary OH groups located at the C-6 position of the galactopyranose residues—are deprotonated, yielding highly nucleophilic alkoxide anions. These activated sites attack the terminal electrophilic carbon atom of the epoxy ring within the EPTAC. The reaction culminates in ring-opening polymerization and the formation of the 2-hydroxy-3-(trimethylammonium)propyl ether of arabinogalactan. At this stage, precise pH control is critical: an increase in pH may promote a side reaction—the formation of a diol from CHPTAC (Figure S2).

As a result of the functionalization of arabinogalactan, a series of samples with different degrees of substitution was obtained, the data on which are presented in Table 1.

An interesting trend is observed as a result of the obtained data. Changing the duration of the process at a reaction mixture pH of 10 does not lead to significant transformations of the arabinogalactan structure. Under these conditions, the maximum DS value (0.02) is achieved already after 24 h, followed by a plateau [51]. The obtained data indicate a low efficiency of CHPTAC conversion to EPTAC during the reaction due to an insufficient alkaline medium [48,51]. With an increase in the pH of the medium to 11 under similar process duration conditions, quaternization intensifies, which makes it possible to achieve the most substituted structures. However, having reached the maximum DS value (0.08) at 30 h, the action of the quaternizing agent slows down due to incomplete conversion of CHPTAC [48], which in turn is reflected in the absence of an increase in the substitution degree. An increase in the pH of the medium to 12 leads to an increase in the substitution degree to 0.19 with a process duration of 30 h. Higher DS values at pH of 12 is due to the active action of the formed epoxide and the substitution of free hydroxyl groups (C-2 and C-3) (Figure 2). However, during the prolonged course of the substitution reaction, the molecule unwinds, which leads to the intensification of hydrolysis reactions along the side glycosidic bonds (1→4) and, accordingly, to a decrease in the degree of substitution to 0.16. Thus, it can be concluded that the reaction mixture environment has a greater influence on the regulation of the degree of substitution [52]. The decrease in product yield (especially at 10–11 pH range) is due not only to the loss of low-molecular-weight fragments during dialysis but also to the degradation of the arabinogalactan backbone and side branches during the reaction, particularly when the reaction time is increased beyond the optimal value (over 30 h). This conclusion is supported by GPC data and degree of substitution analysis: as the reaction time increases beyond 30 h, DS decreases, indicating the predominance of hydrolytic processes.

### 3.2. Process Optimization

In order to find the optimal conditions for the synthesis, a numerical optimization of the process was carried out. The pH (X_1_) and duration of the process (X_2_) were chosen as the variable parameters. The degree of substitution DS (Y_1_) was chosen as the output parameter, by which the efficiency of the conditions for the process of obtaining quaternized arabinogalactan was assessed. The data used for the numerical optimization are presented in Table 1.

The dependences of the output parameters on the process variable factors were approximated by second-order regression equations. The dispersion analysis showed (Table 2) that within the limits of the accepted experimental conditions, the factor pH makes a significant contribution to the total dispersion of the output parameter. This is indicated by the high values of the dispersion relations F for the main effects, also called the efficiencies of influence. The information contained in the P columns (Table 2) is interpreted similarly. The influence of the dispersion source on the output parameter is considered statistically significant if the significance level is less than the specified critical value (0.05).

The results of the analysis of variance (ANOVA) for the mathematical models describing the influence of independent factors on the degree of substitution (Y_1_, DS) and product yield (Y_2_, Yield, wt%). The analysis revealed that the linear effect of factor X_1_ (pH) is statistically most significant for both responses, as evidenced by the highest F-test values (54.74 for Y1 and 35.80 for Y_2_) and significance levels of *p* < 0.001. Factor X_2_ (time) also has a significant effect; however, its contribution to the total variance is significantly smaller.

The nature of the factors’ influence on the target parameters shows significant differences. For the degree of substitution (Y_1_), only the square of factor X_2_ (X_2_^2^, *p* = 0.024) has a significant nonlinear effect, indicating a curvilinear relationship with reaction time. At the same time, for product yield (Y_2_), both quadratic effects (X_1_^2^ and X_2_^2^) are highly significant (*p* = 0.006 and *p* = 0.004, respectively), demonstrating a complex nonlinear response with an optimum. The pairwise interaction effect between factors (X_1_X_2_) was statistically insignificant for both responses (*p* > 0.05).

The resulting models possess high explanatory power. The adjusted coefficient of determination (R^2^adj) is 84.0% for model Y_1_ and 83.9% for model Y_2_, confirming their adequacy and suitability for predicting system behavior within the range of factors studied.

The dependence of DS in arabinogalactan derivatives (Y_1_) on process variables is approximated by the second-order regression Equation (3):

Y_1_ = 1.43089 − 0.29504X_1_ − 0.00400412X_2_ + 0.015X_1_^2^ + 0.000686289X_1_X_2_ − 0.0000343224X_2_^2^ (3)

**Figure 3:**
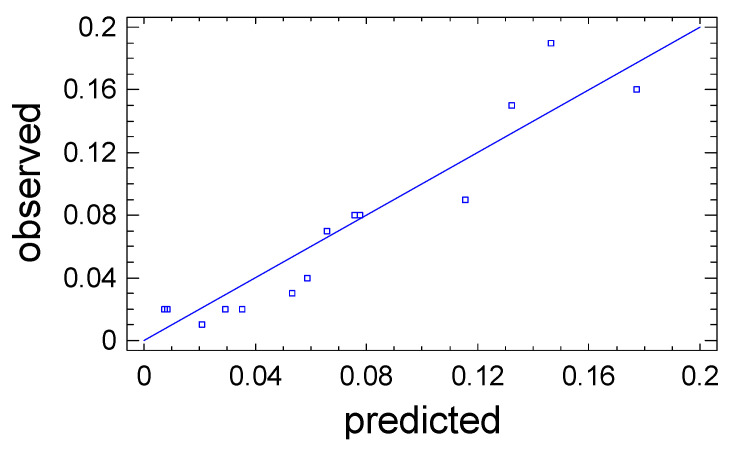
Observation results (points) against the values of the output parameter Y_1_ predicted by the mathematical model.

The predictive properties of Equation (3) are clearly illustrated in Figure 3, where comparative analyses are made of the value of the output parameter Y_1_, which was obtained during the experiment, and the values calculated based on Equation (3). The straight line represents the calculated values of Y_1_, while the points on the graph correspond to the observation data. The close coincidence of the experimental points with the line confirms the high predictive abilities of Equation (3).

**Figure 4:**
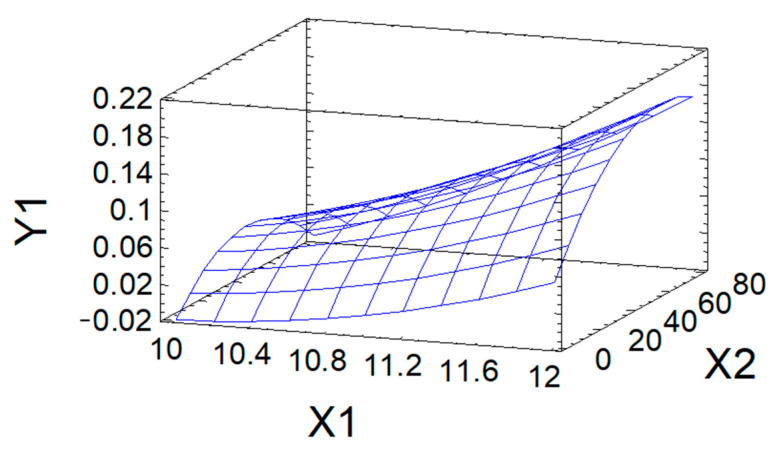
Response surface of DS (Y_1_) in modified AG as a function of pH (X_1_) and process duration (X_2_).

The approximation quality is also characterized by the determination coefficient R^2^_adj_. In the problem under consideration, it has the value R^2^_adj_ = 84.0%, which indicates acceptable approximation quality. This indicates the adequacy of Equation (3) to the observation results and allows it to be used as a mathematical model of the process under study. The mathematical model is used to graphically display the dependence of the output parameter Y_1_ on the variable factors X_1_ and X_2_ in the form of a response surface (Figure 4).

The maximum predicted value of DS (0.21) in the studied region of the factor space is achieved, according to calculations using the mathematical model, at the point corresponding to the following values of variable factors: process pH = 12; process duration of 31.6 h.

The dependence of Yield (wt%) in arabinogalactan derivatives (Y_2_) on process variables is approximated by the second-order regression Equation (4):

Y_2_ = 415.606 − 64.4199X_1_ + 0.462077X_2_ + 3.1X_1_^2^ − 0.0164416X_1_X_2_ − 0.00321872X_2_^2^ (4)

**Figure 5:**
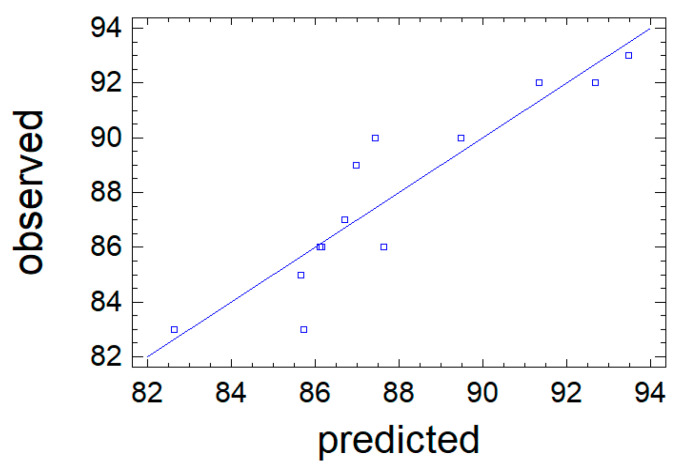
Observation results (points) against the values of the output parameter Y_2_ predicted by the mathematical model 4.

Figure 5 compares the experimental values of the output parameter Y_2_ with the data calculated using Equation (4). The calculated values are shown as a solid line, while the experimental values are shown as dots. The close agreement between the dots and the line demonstrates the high predictive accuracy of Equation (4).

**Figure 6:**
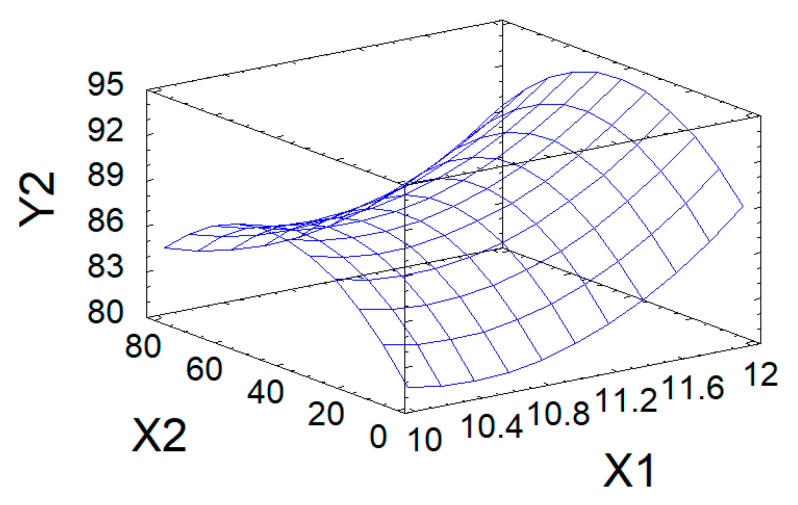
Response surface of Yield (wt%) in modified AG as a function of pH and process duration.

The quality of the approximation was assessed using the adjusted coefficient of determination R^2^adj. The resulting value of 83.9% indicates satisfactory model quality and its adequacy to the experimental data. Thus, Equation (4) can be used to describe the process under study. The dependence of the output parameter Y_2_ on factors X_1_ and X_2_, represented by this model, is visualized as a response surface in Figure 6.

According to calculations using mathematical model 4, the maximum predicted value of the product yield (94.4 wt%) in the studied region of the factor space is achieved with the following values of variable factors: process pH = 12 and process duration of 31.1 h.

### 3.3. Molecular Weight Distribution

**Figure 7:**
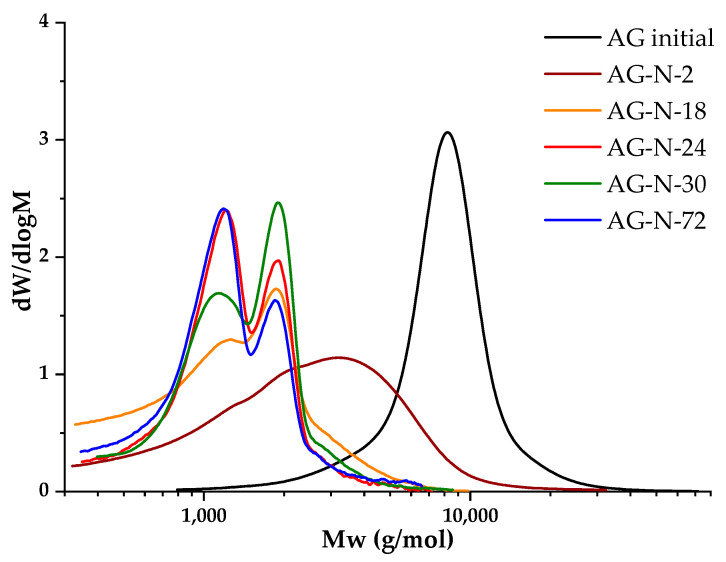
Molecular weight distribution of samples AG initial and quaternized derivatives at different process durations.

Determination of the molecular weight characteristics of the polymer in the study of chemical modification processes helps to identify the corresponding changes in the polymer structure, including the destruction and formation of by-products. In accordance with the literature data [25,26,27,28,29,30,32,33], quaternization in an alkaline medium is carried out at pH ≈ 11–12, 50–70 °C, with a duration of up to 48 h. A series of experiments was carried out at pH of 12, varying the duration of the quaternization process, in order to study the dependence of molecular weights on the duration of the process (Figure 7).

The initial arabinogalactan is a homogeneous polymer with an average molecular weight of 8607 g/mol; the molecular weight distribution is monomodal, the degree of polydispersity is 1.27, which indicates the homogeneity of the sample. When arabinogalactan is modified by quaternization for 2 h, the peak of the molecular weight distribution noticeably broadens, shifts to the low-molecular region, and the value of the average molecular weight decreases to 3104 g/mol (Table 3). With a further increase in the process from 18 to 30 h, two oligomeric fractions are formed, and with an increase in the duration, a redistribution towards the fraction with a lower molecular weight occurs. The degree of substitution increases with an increase in the duration of the process, but after 30 h, a decrease in the studied indicator is observed (Table 3). In parallel with the increase in process time, the side reactions of hydrolysis intensify, which indicates the fact that a process lasting more than 30 h is impractical.

**Figure 8:**
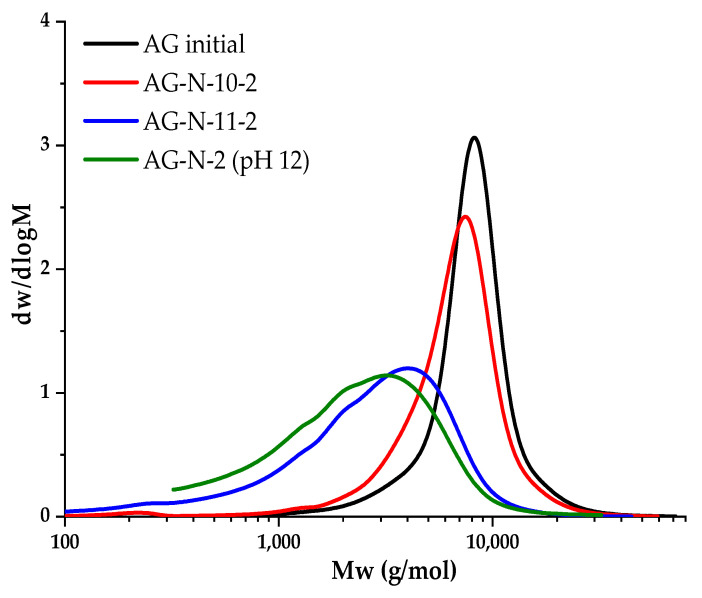
Molecular weight distribution of AG and quaternized AG samples at different pH values.

Since hydrolysis is observed even with a short duration of the quaternization process, it was assumed that the resulting complex is highly active, or the medium is too aggressive. A series of experiments lasting 2 h were carried out, varying the pH of the medium from 10 to 12 to study the relationship between the pH of the solution and the degree of hydrolysis, and these samples were studied by GPC (Figure 8).

Figure 8 shows the shift in peaks to the low-molecular region, as well as a decrease in molecular weight. The largest jump is observed when the pH changes to 11, probably due to the destruction of molecules, as well as an increase in the degree of substitution. At pH of 10, substitution practically does not occur, since the medium is slightly alkaline, the reaction of ring closure of the chlorohydrin group CHPTAC is weakly initiated, the intermediate product is formed to a small extent, and as a result, a small number of epoxy groups are formed. With an increase in pH of 12, the redistribution of peaks is insignificant, but the hydrolysis process is enhanced (Table 4).

### 3.4. Fourier Transform Infrared Spectroscopy of the Arabinogalactans 

**Figure 9:**
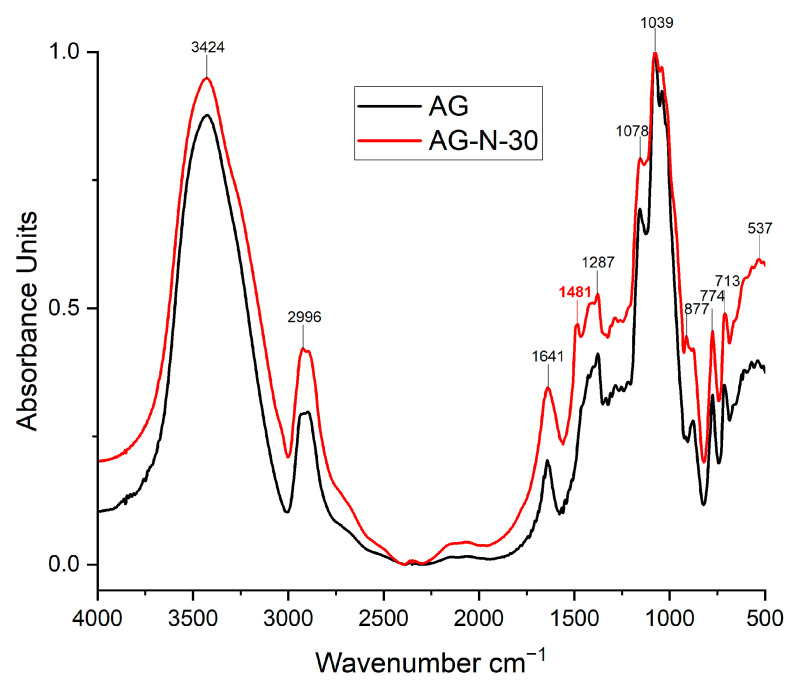
FTIR spectra of the initial and quaternized AGs.

The most notable absorption bands were identified in the structures of the original and quaternized arabinogalactan by FTIR spectroscopy. The most intense characteristic bands are observed in sample AG-N-30, which indicates a high degree of substitution, compared with the other samples (Figure 9).

The broad and strong absorption band at 3424 cm^−1^ corresponds to the characteristic absorption band of the stretching of the hydroxyl groups of the polysaccharide. The vibrations in the region of ~2996 cm^−1^ characterize the stretching of the C-H bonds. The absorption bands at 2996 and 1641 cm^−1^ are the characteristic absorption bands of the deformation vibrations of the C-H and O-H groups of bound water, respectively. In addition, the absorption bands at 1287, 1078, and 877 cm^−1^ are related to the stretching vibration of the C-O bonds of the monosaccharide units [48]. Spectral changes in the 950–800 cm^−1^ range are due to the breakdown of glycosidic bonds, which is caused by the destruction of the native polysaccharide structure. All bands below 800 cm^−1^ cannot be precisely identified, but are attributed to the accepted fact that they correspond to skeletal vibrations of the polysaccharides.

It should be noted that in the quaternized samples, a characteristic peak at 1481 cm^−1^, the intensity of which increases with increasing process duration, corresponds to the C-H bending vibration of the methyl groups of the quaternary ammonium substituents [42,53,54]. In addition, the presence of a small band at 1410 cm^−1^ can also be noted due to the presence of C-N stretching vibrations [30]. The data obtained indicate that in the quaternization process, the degree of substitution increases with increasing time.

### 3.5. Nuclear Magnetic Resonance Measurements

**Figure 10:**
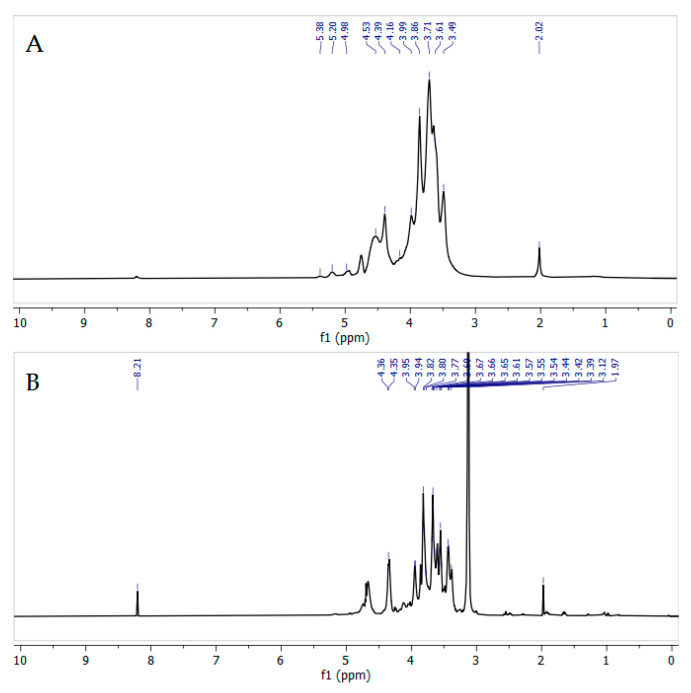
^1^H NMR spectra of initial (**A**) and quaternized (**B**) AGs recorded in D_2_O.

The spectra of the original and modified arabinogalactan contain characteristic signals of the main protons inherent in galactopyranose units (H-1, 4.4–4.6 ppm; H-2, 3.52 ppm; H-3, 3.62 ppm; H-4, 3.9–4.1 ppm; H-5, 3.67–3.88 ppm; H-6, 3.9–4.0 ppm) (Figure 10). Signals of arabinose units in the furanose and pyranose forms are also present (H-1, 5.01–5.31 ppm; H-2, 4.18 ppm; H-3, 3.86 ppm; H-4, 4.1–4.3 ppm; H-5, 3.67–3.88 ppm), which is in good agreement with the literature data [55].

The most noticeable change in the modified AG-N-30 sample is the appearance of a strong signal at 3.24 ppm, corresponding to methyl groups at nitrogen, indicating the incorporation of quaternary ammonium groups into the arabinogalactan structure [49]. The change in the polymer structure of AG, confirmed by the GPC method, is also reflected in the ^1^H NMR spectrum—after modification, the characteristic signals of the protons of the arabinose units, which are in the side chains, disappear. Thus, during the reaction, the glycosidic bonds of both the main chain of AG and the side ones are broken, with the removal of low-molecular fragments.

**Figure 11:**
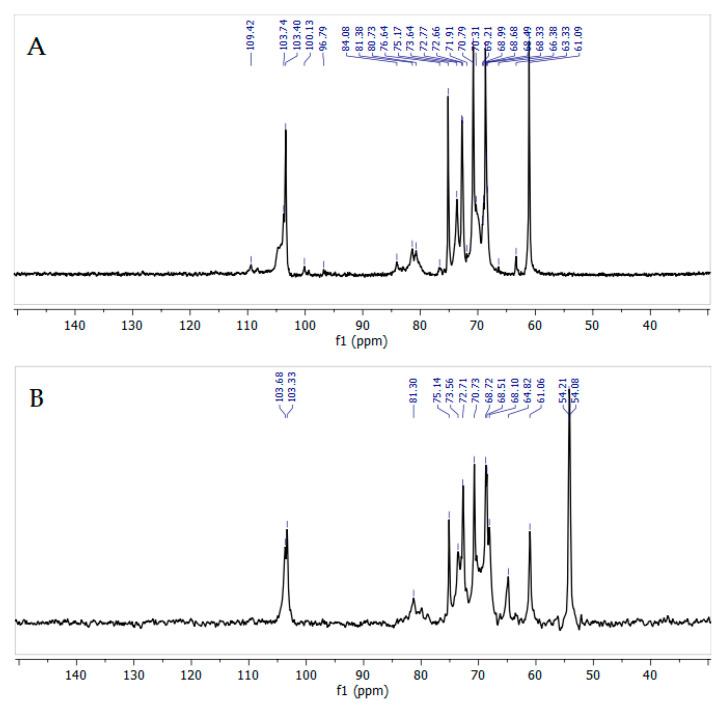
^13^C NMR spectra of initial (**A**) and quaternized (**B**) AGs recorded in D_2_O.

In the ^13^C NMR spectrum of quaternized AG (Figure 11), all the signals corresponding to C1-C6 galactose unit atoms are observed at 100.5, 79.2, 73.7, 72.4, 72.0, and 60.9 ppm, respectively. The main difference in the spectrum of quaternized AG (Figure 11B) is the appearance of a signal at 54.0 ppm, corresponding to the methyl groups of trimethylaminopropyl residues [50].

### 3.6. Thermogravimetric Analysis of the Arabinogalactans

**Figure 12:**
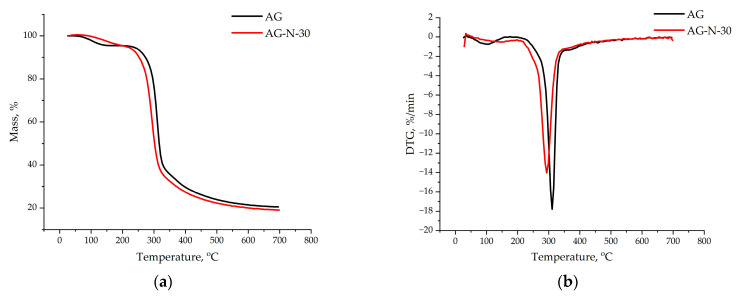
TGA (**a**) and DTG (**b**) thermal degradation profiles of initial and quaternized AG samples.

The thermogravimetric analysis showed (Figure 12) that the thermal decomposition of the original arabinogalactan and quaternized arabinogalactan can be divided into three main stages: (1) drying, (2) removal of volatile substances, and (3) formation of coke residue.

The mass loss of the initial arabinogalactan begins at 87 °C, corresponding to the evaporation of bound water in the range of 80–100 °C. In the quaternized sample, water loss occurs in the range of 100–150 °C, which indicates stronger water–polymer interactions due to the addition of new functional groups. The peak of the main decomposition in quaternized arabinogalactan is shifted to a lower temperature region due to the degradation of attached groups. The residual mass of the quaternized arabinogalactan is 1.5% lower than that of the initial sample; this difference is due to the different functional composition. Smooth decomposition of molecules occurs due to the elimination of volatile functional groups.

### 3.7. Antioxidant Activity of the Initial AG and Its Quaternized Derivatives

Determining the antioxidant potential of any new material is a key factor in determining its applicability for biological purposes [51]. Based on the structure–property relationship, native polysaccharides may have relatively weak biological activity compared to endogenous antioxidants (Vitamin C). However, chemical modification of the molecule promotes the formation of new derivatives, and the structural transformations occurring in this process contribute to the change in the physicochemical and biological properties of polysaccharides [56]. Quaternization is one such method that is used to improve the biological functionalization of polymer macromolecules [26].

**Figure 13:**
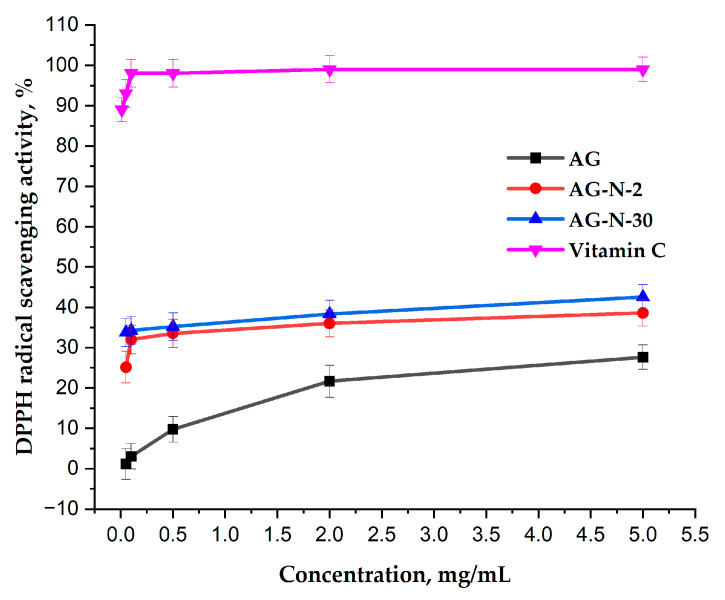
Absorption capacity of DPPH radicals by aqueous solutions of arabinogalactan and its quaternized derivatives.

In this study, the DPPH free radical scavenging ability of native arabinogalactan and its two quaternized derivatives with different degrees of substitution (0.04 and 0.19) was tested. The obtained data (Figure 13) indicate that with the initial structure, arabinogalactan, although exhibiting antioxidant capacity, is quite weak, reaching a maximum of ~25% at a polysaccharide concentration of 5 mg/mL and gradually reaching a plateau. At the same time, quaternized derivatives exhibit a greater ability to inhibit free radicals over the entire range of polysaccharide solution concentrations, reaching a maximum of ~42%.

The increased antioxidant potential of arabinogalactan is directly related to the fact that positively charged groups can alter the electron density in adjacent regions of the polymer chain, which, as a result, can facilitate more efficient proton transfer or stabilization of the polysaccharide radical formed during the reaction [26,57].

Following the DPPH radical scavenging reaction, no polysaccharide degradation processes were observed: both the distribution and the polydispersity remained unchanged. This indicates that the scavenging of the 2,2-diphenyl-1-picrylhydrazyl radical does not induce hydrolysis of the native and modified arabinogalactan backbone (Figures S3 and S4).

Based on the data obtained, it can be noted that quaternized arabinogalactan with a high degree of substitution can be used as a preventive antioxidant involved in the inhibition of free radical chain reactions.

## 4. Conclusions

Quaternized derivatives of arabinogalactan were successfully synthesized by the interaction of the polysaccharide and a system based on the quaternary ammonium reagent CHPTAC, catalyzed by an aqueous solution of NaOH. By varying the synthesis conditions, specifically pH of the reaction mixture and the duration of the process, samples with a high substitution degree (0.19) were obtained. According to mathematical calculations, the optimal conditions for the modification of arabinogalactan are pH of 12, with a process duration and temperature of 31.6 h and 50 °C, respectively. All the obtained derivatives were characterized by gel permeation chromatography to demonstrate the structural changes occurring during the quaternization reaction. In addition, the inclusion of quaternary ammonium groups was confirmed by FTIR and NMR spectroscopy. When evaluating the antioxidant activity, it was found that quaternized arabinogalactan derivatives exhibit increased values of free radical inhibitory capacity compared to the native sample, which opens up potential opportunities for their use in the food and pharmacological fields as biologically active additives.

## Figures and Tables

**Table 1:** Data on the degree of substitution in modified arabinogalactan samples depending on the variable parameters (pH of the medium and duration of the process).

Samples	pH (X_1_)	Time, h (X_2_)	DS (Y_1_)	Yield, wt% (Y_2_)
AG initial			0	-
AG-N-2	12	2	0.04 ± 0.01	90 ± 1
AG-N-18	12	18	0.09 ± 0.01	92 ± 1
AG-N-24	12	24	0.15 ± 0.02	93 ± 1
AG-N-30	12	30	0.19 ± 0.02	94 ± 2
AG-N-72	12	72	0.16 ± 0.02	92 ± 1
AG-N-11-2	11	2	0.02 ± 0.00	83 ± 1
AG-N-11-18	11	18	0.03 ± 0.00	86 ± 1
AG-N-11-24	11	24	0.07 ± 0.01	89 ± 2
AG-N-11-30	11	30	0.08 ± 0.01	86 ± 1
AG-N-11-72	11	72	0.08 ± 0.01	85 ± 1
AG-N-10-2	10	2	0.01 ± 0.00	82 ± 1
AG-N-10-18	10	18	0.01 ± 0.00	83 ± 1
AG-N-10-24	10	24	0.02 ± 0.00	87 ± 1
AG-N-10-30	10	30	0.02 ± 0.00	90 ± 2
AG-N-10-72	10	72	0.02 ± 0.00	86 ± 1

**Table 2:** Results of the analysis of variance.

Sources of Dispersion	DS (Y_1_)		Yield, wt% (Y_2_)	
	Dispersion Relations, F	Levels of Significance, P	Dispersion Relations, F	Levels of Significance, P
X_1_	54.74	0.0000	35.80	0.0002
X_2_	13.03	0.0057	5.65	0.0414
X_1_^2^	1.25	0.2920	12.67	0.0061
X_1_ X_2_	4.29	0.0683	0.58	0.4648
X_2_^2^	7.40	0.0236	15.41	0.0035
R^2^_adj_	84.0		83.9	

**Table 3:** Molecular weight characteristics and DS of AG and AG-N samples depending on the process duration.

Samples	M_p_ (g/mol)	M_n_ (g/mol)	M_w_ (g/mol)	PDI	DS
AG initial	8253	6799	8607	1.27	0
AG-N-2	3090	1604	3104	1.93	0.04
AG-N-18	1865	1005	1510	1.50	0.09
AG-N-24	1204	1123	1412	1.26	0.15
AG-N-30	1897	1212	1540	1.27	0.19
AG-N-72	1182	1055	1380	1.31	0.16

**Table 4:** Molecular weight characteristics of AG and AG-N samples depending on the pH of the medium.

Samples	M_p_ (g/mol)	M_n_ (g/mol)	M_w_ (g/mol)	PDI
AG initial	8253	6799	8607	1.27
AG-N-10-2	7448	4189	7303	1.74
AG-N-11-2	3922	1322	3438	10.68
AG-N-2 (pH 12)	3090	1604	3104	10.93

## Data Availability

All data generated during this study are included in the article.

## Supplementary Materials

The following supporting information can be downloaded at: https://www.mdpi.com/article/10.3390/polym18020148/s1.
