# Supplementary material for: New Polycationic Arabinogalactan Derivatives with the CHPTAC System: Structure, Properties and Antioxidant Activity
Source: Polymers (Basel). 2026 Jan 6;18(2):148. doi: 10.3390/polym18020148 (PMC12845099; doi:10.3390/polym18020148)
Supplement: Supplementary file 1 [file polymers-18-00148-s001.zip › polymers-4022904-supplementary.pdf]

Supplementary materials

New Polycationic Arabinogalactan Derivatives with the CHPTAC System: Structure, Properties and Antioxidant Activity

Maria V. Sereda [1,2], Yuriy N. Malyar [1,2,*], Valentina S. Borovkova [1,2], Alexander S. Kazachenko [1,2,3]

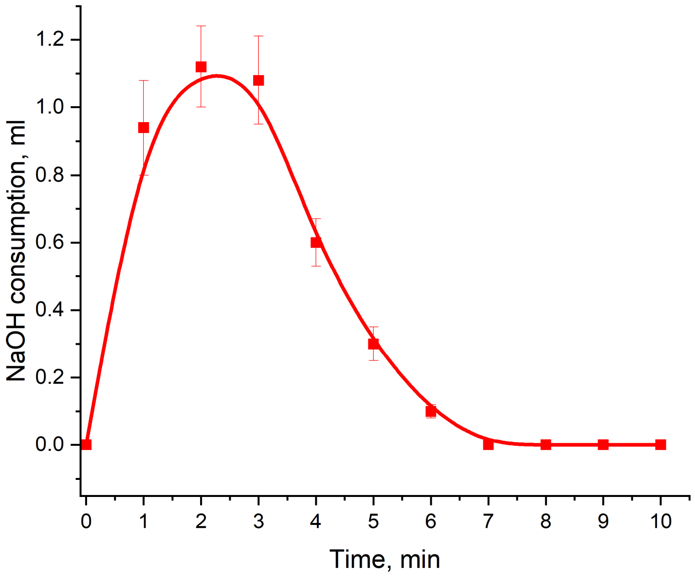

Figure S1. Consumption of NaOH (mL) during the quaternization of AG

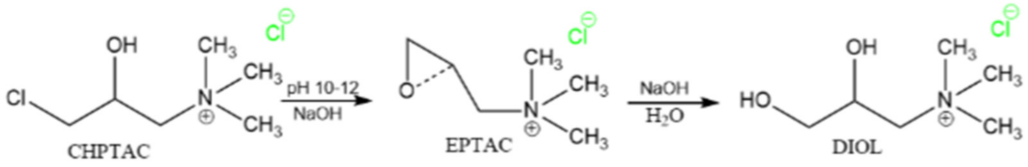

Figure S2. CHPTAC conversion to EPTAC and diol.

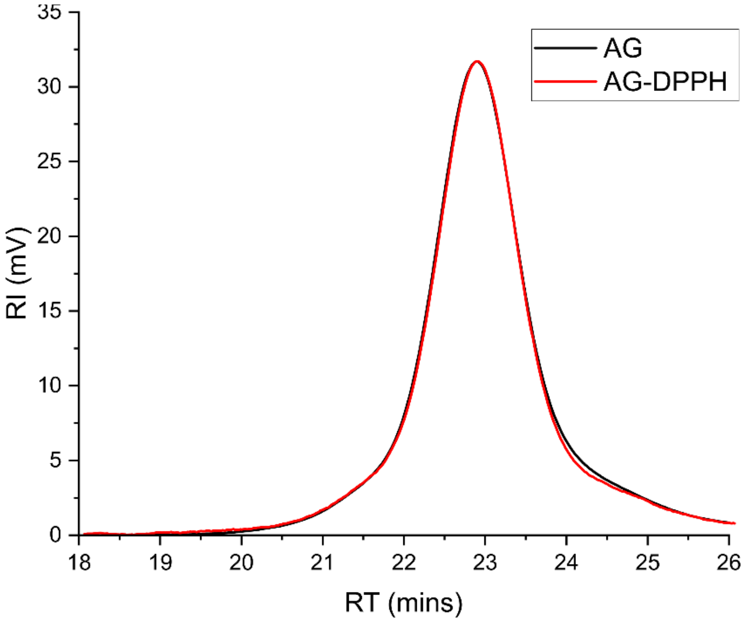

Figure S3 - GPC chromatograms of AG before and after interaction with DPPH

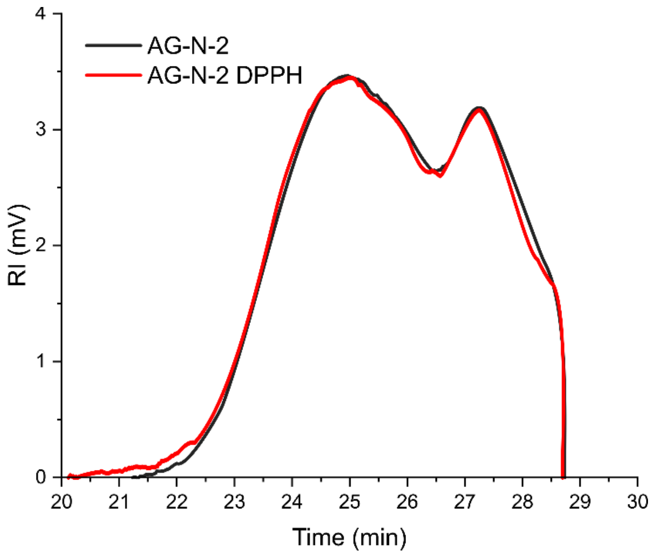

Figure S4 - GPC chromatograms of AG-N-2 before and after interaction with DPPH
